# Skeletal Editing by Tip-Induced Chemistry

**DOI:** 10.1021/jacs.5c16196

**Published:** 2025-11-20

**Authors:** Shantanu Mishra, Valentina Malave, Rasmus Svensson, Henrik Grönbeck, Florian Albrecht, Diego Peña, Leo Gross

**Affiliations:** † Department of Physics, 11248 Chalmers University of Technology, 412 96 Göteborg, Sweden; ‡ IBM Research Europe − Zurich, 8803 Rüschlikon, Switzerland; § Center for Research in Biological Chemistry and Molecular Materials, and Department of Organic Chemistry, 16780 University of Santiago de Compostela, 15782 Santiago de Compostela, Spain; ∥ Competence Centre for Catalysis, 11248 Chalmers University of Technology, 412 96 Göteborg, Sweden; ⊥ Oportunius, Galician Innovation Agency, 15702 Santiago de Compostela, Spain

## Abstract

Skeletal editing of cyclic molecules has garnered considerable attention in the context of drug discovery and green chemistry with notable examples in solution-phase synthesis. Here, we extend the scope of skeletal editing to the single-molecule scale. We demonstrate tip-induced oxygen deletion and ring contraction of an oxygen-containing seven-membered ring on bilayer NaCl films to generate molecules containing the perylene skeleton. The products were identified and characterized at the atomic scale by atomic force microscopy and scanning tunneling microscopy. Insights into the reaction mechanisms were obtained by density functional theory calculations. Our work expands the toolbox of tip-induced chemistry for single-molecule synthesis.

## Graphical abstract

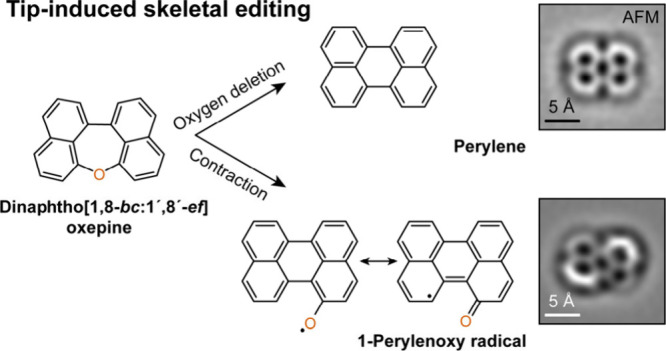

Skeletal editing^1^ refers to the modification of the cyclic skeleton of an organic molecule by inserting, deleting, or swapping individual atoms at precise locations at late stages of a synthetic sequence. The process is schematically depicted in Scheme 1, where it is contrasted with peripheral editing. Skeletal editing is poised to be of great importance in medicinal chemistry,^2,3^ where precise edits to the molecular skeleton without the design of new synthetic routes from scratch could dramatically speed up drug discovery. Skeletal editing has also been employed to modify polymer backbones,^4^ which may open avenues for upcycling of plastics. The current repertoire of skeletal editing in solution-phase chemistry includes (a) deletion^5–7^ and insertion^8–10^ of C, N, and O atoms; (b) swapping^11–13^ of ^12^C with ^13^C, N, and O; and (c) ring contraction^14^ and expansion.^15^ In this context, tip-induced chemistry,^16,17^ wherein voltage pulses applied by a scanning probe tip are used to induce chemical reactions of molecular species adsorbed on surfaces, holds distinct advantages. Atomic-scale structural and electronic characterizations of molecular species can be performed by scanning tunneling microscopy (STM) and atomic force microscopy (AFM). Furthermore, the solvent-free, cryogenic, and ultrahigh-vacuum conditions facilitate generation (sometimes with high selectivity^18^), stabilization, and characterization of reactive intermediates and products, which can yield mechanistic insights into chemical reactions. Tip-induced chemistry has been successfully employed to generate molecules by peripheral editing, such as through dissociation of C–Cl,^18,19^ C–Br,^20,21^ C–I,^22^ C–C,^23,24^ C–H,^25,26^ C–O,^27^ N–H,^28^ and N–N^29^ bonds, attachment of foreign molecules,^30^ and skeletal rearrangement.^31,32^ Here, we demonstrate tip-induced skeletal editing (Scheme 1). From dinaphtho­[1,8-*bc*:1′,8*′-ef*]­oxepine (**DNO**), we generated different molecules with the perylene skeleton by oxygen deletion, and ring contraction and oxygen migration.

**Scheme 1:**
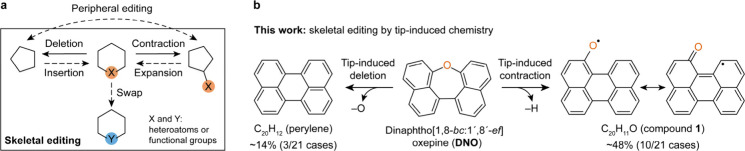
(a) Illustration of Skeletal and Peripheral Editing;^a^ (b) Scheme Showing the Generation of the Two Majority Products by Tip-Induced Skeletal Editing of **DNO**

a The solid arrows denote the skeletal editing reactions observed in this work.

**DNO** was synthesized by solution-phase chemistry (Schemes S1–S5 and Figures S1–S7) and deposited on a single-crystal Cu(111) surface partially covered by bilayer NaCl films (Figure S8). Figure 1a shows an STM image of the surface, revealing isolated **DNO** molecules, along with coadsorbed carbon monoxide (CO) molecules and a minority of third-layer NaCl islands. Nearly all **DNO** molecules (66 out of 67 imaged **DNO** molecules) on NaCl adopted an adsorption conformation wherein the oxygen atom points toward the surface (denoted O-down). Figures 1b–d show STM and AFM images of an O-down **DNO** molecule. In AFM imaging at large tip heights (Figure 1c), only two benzenoid rings of the molecule are visible, and in imaging at small tip heights, all four benzenoid rings become visible (Figure 1d). Related to the adsorption geometry, the oxygen atom is not visible. The conspicuous streaks in the STM and AFM images likely result from conformational switching of the molecule under the influence of the tip, related to the relative tilting of the benzenoid rings. Rarely, we found **DNO** molecules on NaCl wherein the oxygen atom points away from the surface (O-up, Figures 1e–g). In this case, the oxygen atom, being the atomic species closest to the tip, appears bright in AFM imaging (that is, with an increased frequency shift Δ*f*). Density functional theory (DFT) calculations of **DNO** on NaCl/Cu(111) predicted the O-down conformation to be ∼0.1 eV more stable than the O-up conformation, in line with the experimentally observed predominance of the O-down species. Figure S9 shows further measurements on **DNO**.

**Figure 1:**
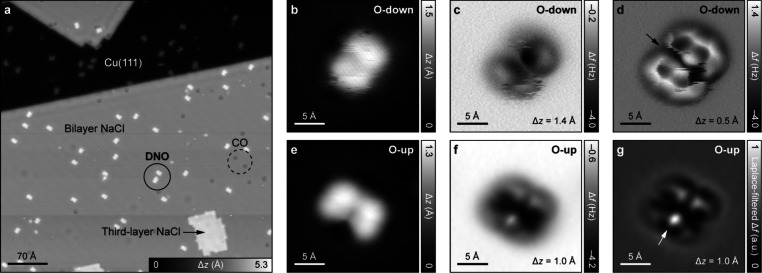
Characterization of **DNO** on NaCl/Cu(111). (a) STM overview image of the sample. (b–d) STM (b) and AFM (c, d) images of an O-down DNO molecule. (e–g) STM (e), AFM (f), and the corresponding Laplace-filtered AFM (g) images of an O-up **DNO** molecule. The arrows in (d, g) indicate the location of the oxygen atom. Scanning parameters for STM images: *V* = 0.2 V, *I* = 0.5 pA. Open feedback parameters for AFM images: *V* = 0.2 V, *I* = 0.5 pA on NaCl. Δ*z* denotes the tip-height offset, with positive (negative) values indicating tip retraction (approach) from the set-point conditions.

Voltage pulses ranging between 4.9 and 5.1 V were applied to individual **DNO** molecules by the tip of the STM/AFM system to trigger intramolecular chemical reactions (Methods and Figure S10). From 99 voltage pulses applied to 28 **DNO** molecules, we observed 21 molecules that underwent a reaction on the surface, resulting in 6 unique species. In unsuccessful cases, **DNO** molecules were either displaced on NaCl and remained intact, or were not located, presumably being picked up by the tip or displaced from NaCl onto Cu(111). Out of the 21 reacted molecules, the majority (16/21) corresponded to molecules with the perylene skeleton (constituting 4 species), namely, perylene (3/21), 1-perylenoxy radical (compound **1**; 10/21), perylenyl radical (compound **2**; 2/21), and didehydroperylene (compound **3**; 1/21). In cases where the oxygen atom was removed from **DNO** (as in perylene, **2**, and **3**), the oxygen atom was not found on the surface, likely because of its desorption to the gas phase or diffusion on NaCl followed by adsorption on Cu(111). Here, we focus on the characterization of perylene and **1**, the major products of atom deletion and ring contraction, respectively. The characterizations of **2** and **3** are reported in Figure S11, and data on the remaining two species (5/21 cases) are shown in Figure S12.

Figure 2a shows an AFM image of perylene generated by the tip-induced removal of an oxygen atom from **DNO**. STM imaging at bias voltage *V* = 1.8 V (Figure 2b) revealed the lowest unoccupied molecular orbital (LUMO) density of perylene, in agreement with calculations (Figure S13). We next focused on the characterization of **1**, which maintains an oxygen atom attached to the perylene skeleton (Scheme 1). At the outset, we note that **1** is found in an anionic charge state on the surface (denoted as **1** ^**–**^). This is experimentally inferred from the scattering of the NaCl/Cu(111) interface state by the molecule (Figure S14) and supported by Bader charge analysis of the molecule adsorbed on bilayer NaCl/Cu(111), yielding a transfer of 0.81 electrons from the surface to **1**. In contrast to charge-neutral **1** (denoted **1** ^**0**^) being an open-shell species, **1** ^**–**^ is closed-shell (Figure S15). The optimized C–O bond length of **1** ^**–**^ on bilayer NaCl/Cu(111) is calculated to be 1.29 Å. To contextualize this value in terms of the single- or double-bond character of the C–O bond, we carried out gas-phase calculations (Table S1) of cyclohexanone and phenol, which contain C­(*sp* ^2^)–O double and single bonds, respectively. From these calculations, we obtained C–O bond lengths of 1.23 Å (cyclohexanone) and 1.38 Å (phenol). The comparison of the C–O bond lengths thus indicates that **1** ^**–**^ is best described as a resonance hybrid of structures containing C–O single and double bonds (Scheme 1). Figures 2c–e show the STM and AFM images of **1** ^**–**^. In AFM imaging of **1** ^**–**^ (Figures 2d,e), the benzenoid ring bonded to an oxygen atom (highlighted by the arrow in Figure 2d) appears notably dark, whereas two peripheral benzenoid rings that lie diagonally opposite each other appear bright. This observation agrees with the DFT calculation of **1** ^**–**^ on bilayer NaCl/Cu(111) (Figure 2f), which shows a nonplanar adsorption conformation of **1** ^**–**^ with the oxygen atom pointing toward the surface and an upward tilt of the two peripheral benzenoid rings. Figures 2g,h show the STM and AFM images of **1** ^**–**^ adsorbed next to a **DNO** molecule that rendered **1** ^**–**^ stable for imaging at elevated voltages. We could therefore measure the highest occupied molecular orbital (HOMO) density of **1** ^**–**^ at −1.0 V (Figure 2i), which exhibited good agreement with the calculated HOMO of **1** ^**–**^ (Figure 2j).

**Figure 2:**
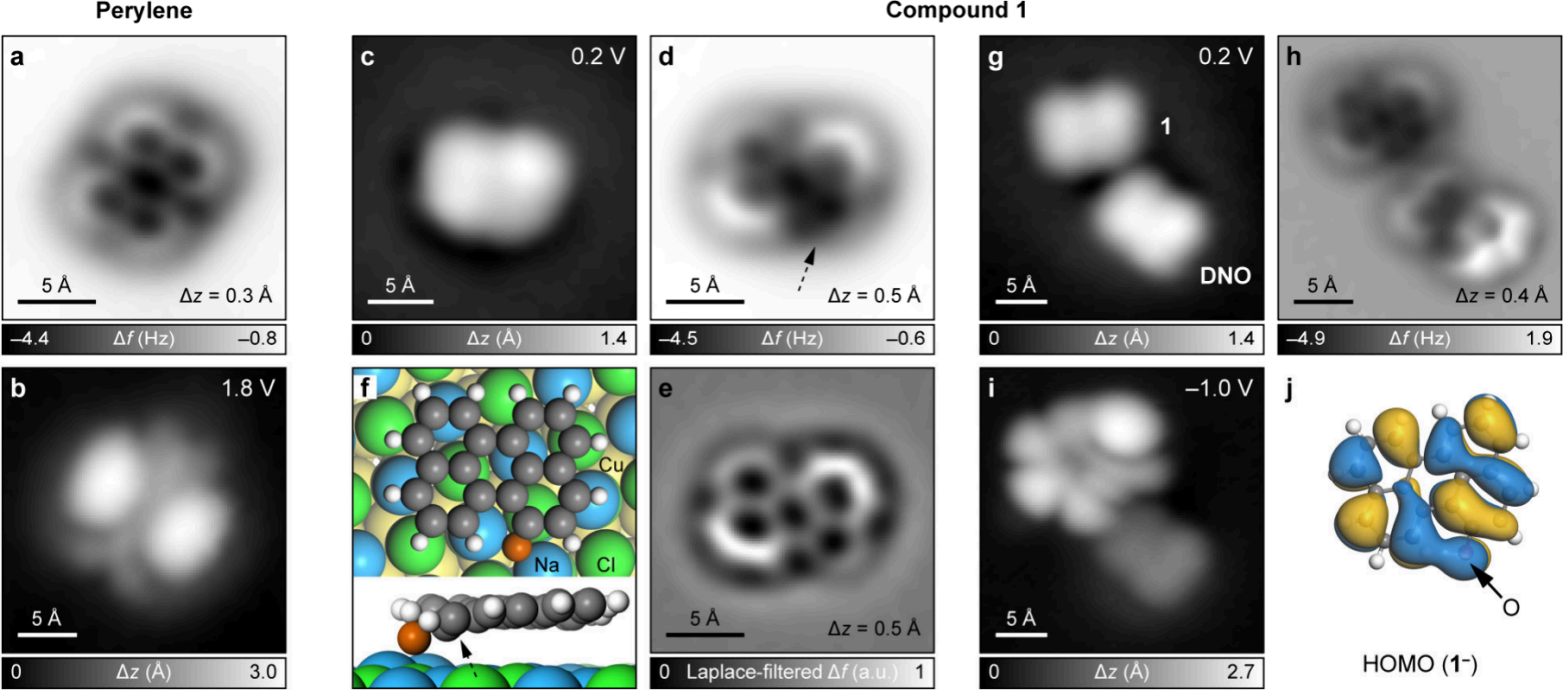
Structural and electronic characterizations of perylene and compound **1**. (a) AFM image of perylene. (b) STM image of perylene showing its LUMO density. (c–e) STM (c), AFM (d), and corresponding Laplace-filtered AFM (e) images of **1** ^–^. (f) Top and side views of the DFT-calculated adsorption geometry of **1** ^–^ on bilayer NaCl/Cu(111). The oxygen atom adsorbs bridging two Na^+^ ions, which show an outward relaxation of ∼0.5 Å. The arrows in (d, f) indicate the six-membered ring bonded to the oxygen atom. C, H, and O atoms are colored gray, white, and orange, respectively. (g, h) STM (g) and AFM (h) images of **1** ^–^ adsorbed next to a **DNO** molecule. (i) Corresponding STM image showing the HOMO density of **1** ^–^. (j) DFT-calculated HOMO of **1** ^–^ (isosurface: 0.01 *a*_0_^–3/2^, *a*_0_ denotes the Bohr radius). Scanning parameters for STM images: *I* = 0.15 pA (b), *I* = 0.5 pA (c), and *I* = 0.3 pA (g, i). Open feedback parameters for AFM images: *V* = 0.2 V, *I* = 0.5 pA on NaCl.

Insights into the mechanisms of skeletal editing reactions were obtained by DFT calculations of the reaction landscape. Figure 3 summarizes the results and highlights the relevant reaction paths and energy barriers involved in the generation of perylene, **1**, and **2**. Given the weak molecule–surface interactions on NaCl, we focus on elucidating the reaction mechanisms in the gas phase (solid lines in Figure 3). Starting from **DNO**, the first step involves the formation of a C–C bond resulting in a central six-membered ring, along with migration of the oxygen atom to a neighboring C–C bridge site (intermediate **Int1**, activation energy Δ*E* ^‡^ = 2.70 eV). Three reaction paths are possible after the first step. First, the oxygen atom in **Int1** can be eliminated (Δ*E* ^‡^ = 3.85 eV), leading to the generation of perylene. Second, the oxygen atom in **Int1** can migrate to the next C–C bridge site (Δ*E* ^‡^ = 1.02 eV), leading to intermediate **Int2**, from where the oxygen atom can once again be eliminated (Δ*E* ^‡^ = 3.44 eV) to generate perylene. Third, a rearrangement reaction can occur, resulting in the formation of intermediate **Int3**. The reaction is exothermic and associated with Δ*E* ^‡^ = 1.20 eV. From **Int3**, either the hydroxy group can be removed to generate **2**, which requires a large Δ*E* ^‡^ of 4.81 eV, or the hydrogen atom can be removed from the hydroxy group to generate **1** (Δ*E* ^‡^ = 3.28 eV). We also calculated the potential energy landscape on bilayer NaCl/Cu(111) (dashed lines in Figure 3), where the relative energies of all intermediates and products are similar to those in the gas phase, apart from **1**, which is stabilized on the surface (Figure 2f). We note that intermediates **Int1**–**Int3** were not observed in the experiments. The gas-phase calculations in Figure 3 were performed assuming a neutral charge state of the reactant, intermediates, and products. We also performed gas-phase calculations assuming a global anionic state, which is a possibility given the large positive applied voltage pulses that could cause transient charging of the species. As shown in Figure S16, the reaction mechanisms in the anionic state are similar, but there is a notable lowering of all activation barriers compared to the neutral case. The importance of using the insulating NaCl surface to reduce molecule–surface interactions and facilitate skeletal editing is emphasized by the fact that we were unable to perform skeletal editing on Cu(111), where chemisorption of intermediates or competing reactions were observed instead (Figure S17).

**Figure 3:**
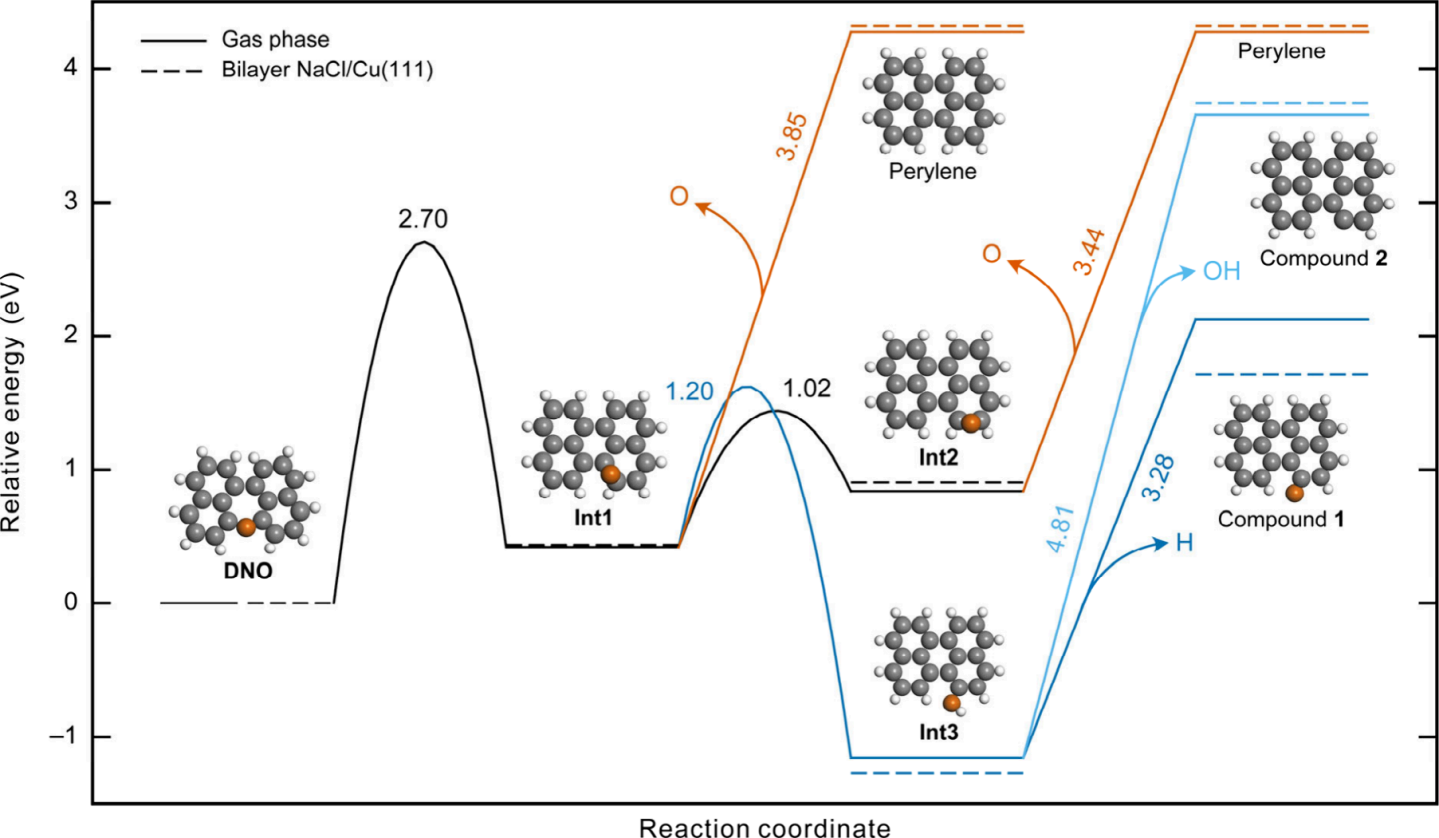
Ab initio potential energy landscape in the gas phase (solid lines) and on bilayer NaCl/Cu(111) (dashed lines) for skeletal editing of DNO. The optimized gas-phase geometries of DNO, intermediates, and products are also shown. The numbers adjacent to the curves denote absolute gas-phase activation energies in eV. Note that for the reactions **Int1** and **Int2** → perylene and **Int3** → compounds **1** and **2**, energy differences coincide with activation energies. For on-surface calculations, gas-phase barrier heights are used, and the dissociated O, H, and OH species are assumed to desorb.

In conclusion, we demonstrate skeletal editing via tip-induced chemistry. Voltage pulses applied by a scanning probe tip to dinaphtho­[1,8-*bc*:1′,8*′-ef*]­oxepine molecules adsorbed on NaCl resulted in two distinct skeletal editing reactions: atom deletion and ring contraction. The former predominantly led to the generation of perylene, whereas the latter led to the generation of 1-perylenoxy radical. Experimental characterization of the products was performed by STM and AFM, and a detailed mechanistic understanding of the intramolecular reactions was obtained from DFT calculations. Future directions could involve exploring how heteroatoms of the same group (such as sulfur) affect the reaction landscape and yields, and performing the challenging atom insertion and swap edits. Obtaining selectivity of the different skeletal editing reactions by tip-induced chemistry would be another goal. Tip-induced skeletal editing may be used for precise local modification of heteroatom-containing carbon nanostructures to imprint electronic^33–36^ and magnetic^37^ functionalities.

## Supplementary Material



## Notes

Experimental and theoretical methods, solution synthesis of **DNO**, ^1^H and ^13^C NMR spectra, mass spectroscopy data, STM and AFM data, and additional calculations (PDF)
