# Supplementary material for: Skeletal Editing by Tip-Induced Chemistry
Source: J Am Chem Soc. 2025 Nov 20;147(48):44055–9. doi: 10.1021/jacs.5c16196 (PMC12679623; doi:10.1021/jacs.5c16196)
Supplement: Supplementary file 1 [file ja5c16196_si_001.pdf]

# Supporting Information

## Skeletal editing by tip-induced chemistry

Shantanu Mishra,[1,2] Valentina Malave,[3] Rasmus Svensson,[1,4] Henrik Grönbeck,[1,4] Florian Albrecht,[2] Diego Peña,[3,5] Leo Gross[2]

[1]Department of Physics, Chalmers University of Technology, 412 96 Göteborg, Sweden

[2]IBM Research Europe – Zurich, 8803 Rüschlikon, Switzerland

[3]Center for Research in Biological Chemistry and Molecular Materials, and Department of Organic Chemistry, University of Santiago de Compostela, 15782 Santiago de Compostela, Spain

[4]Competence Centre for Catalysis, Chalmers University of Technology, 412 96 Göteborg, Sweden

[5]Oportunius, Galician Innovation Agency (GAIN), 15702 Santiago de Compostela, Spain

**Contents**

1. Methods Page S2

2. Supporting data Page S11

3. References Page S18

# 1. Methods

## 1.1. Solution synthesis and characterization

Starting materials were purchased reagent grade from TCI or Sigma-Aldrich and used without further purification. $CH_2Cl_2$ was dried using a MBraun SPS-800 Solvent Purification System. All reactions were carried out in flame-dried glassware under an inert atmosphere of purified Ar using Schlenk techniques. Deuterated solvents were purchased from Acros Organics. Thin-layer chromatography (TLC) was performed on Silica Gel 60 F-254 plates (Merck) and chromatograms were visualized with UV light (254 and 365 nm) and/or stained with Hanessian's stain. Column chromatography was performed on silica gel (40-60 µm). $^1H$ and $^{13}C$ NMR spectra were recorded at 300 MHz ($^1H$) with Varian Mercury 300 instrument, and at 500 MHz ($^1H$) and 125 MHz ($^{13}C$) MHz with Bruker 500 instrument. Mass spectra, using the atmospheric pressure chemical ionization (APCI) method, were recorded on a Bruker MicroTOF spectrometer.

Oxepin **DNO** was obtained following the route shown in Scheme S1, which is based on a previously reported procedure.[1]

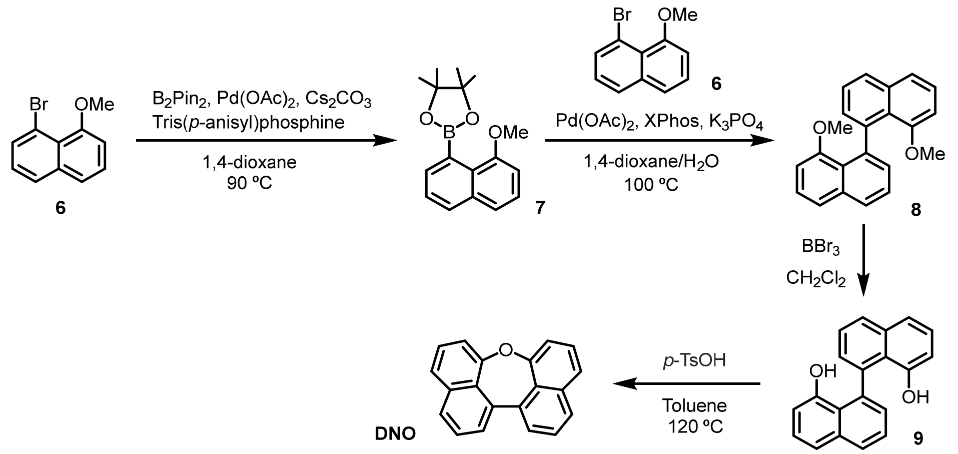


**Scheme S1**. Synthetic route toward the oxepin **DNO**.

### Synthesis of compound 7

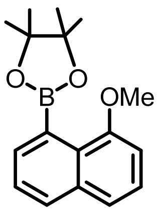


**Scheme S2.** Chemical structure of compound **7**.

To a flame-dried Schlenk flask, compound **6** (100 mg, 0.42 mmol), bis(pinacolato)diboron ($B_2Pin_2$) (160.7 mg, 0.63 mmol), $Cs_2CO_3$ (412.3 mg, 1.26 mmol), $Pd(OAc)_2$ (9.5 mg, 0.04 mmol) and tris(4-methoxyphenyl)phospine (29.7 mg, 0.08 mmol) were added. Then, 1,4-dioxane (6.5 mL) was added, and the reaction mixture was stirred at 90 ºC for 5 h. After reaction completion (monitored by TLC), the reaction mixture was cooled to room temperature and concentrated under reduced pressure to remove

the solvent. The crude residue was purified by column chromatography ($SiO_2$; hexane:$CH_2Cl_2$ 3:2) to afford compound **7** (107 mg, 89%) as a white solid.

**$^1$H NMR** (500 MHz, $CDCl_3$, Fig. S1) δ: 7.79 (dd, *J* = 8.1 Hz, 1H), 7.52 (dd, *J* = 6.8 Hz, 1H), 7.47 – 7.40 (m, 2H), 7.38 – 7.34 (m, 1H), 6.84 (dd, *J* = 7.6 Hz, 1H), 4.01 (s, 3H), 1.45 (s, 12H) ppm. **$^{13}$C NMR** (125 MHz, $CDCl_3$, Fig. S2) δ: 155.77 (C), 134.31 (C), 130.18 (CH), 128.74 (CH), 127.57 (C), 125.74 (CH), 121.17 (CH), 104.75 (CH), 83.72 (C), 55.70 ($CH_3$), 25.28 ($CH_3$) ppm. **MS (APCI)** *m/z* (%): 283 (M-1, 100) (MS denotes mass spectrum).

**Synthesis of compound 8**

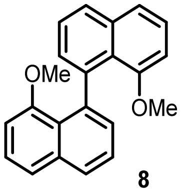


**8**

**Scheme S3.** Chemical structure of compound **8**.

To a round-bottom flask, compound **7** (107.0 mg, 0.38 mmol), compound **6** (50.0 mg, 0.21 mmol), $K_3PO_4$ (134.3 mg, 0.63 mmol), $Pd(OAc)_2$ (9.5 mg, 0.04 mmol) and XPhos (20.1 mg, 0.04 mmol) were added. The solids were dissolved in a mixture of 1,4-dioxane/$H_2O$ (1:1, 4.0 mL), and the resulting solution was heated at 100 ºC for 5 h. After reaction completion (monitored by TLC), the reaction mixture was cooled to room temperature and diluted with $CH_2Cl_2$. The organic phase was washed with water, dried over $Na_2SO_4$, filtered, and concentrated under reduced pressure. The crude residue was purified by column chromatography ($SiO_2$; hexane:$CH_2Cl_2$ 1:1) to afford compound **8** (61.0 mg, 91%) as a white solid.

**$^1$H NMR** (500 MHz, $CDCl_3$, Fig. S3) δ: 7.78 (dd, *J* = 8.2 Hz, 2H), 7.50 (d, *J* = 8.1 Hz, 2H), 7.47 – 7.42 (m, 2H), 7.34 (t, *J* = 7.9 Hz, 2H), 7.21 (dd, *J* = 7.0 Hz, 2H), 6.66 (d, *J* = 7.5 Hz, 2H), 3.05 – 3.03 (m, 6H) ppm. **$^{13}$C NMR** (125 MHz, $CDCl_3$, Fig. S4) δ: 157.42 (C), 142.19 (C), 134.86 (C), 126.84 (CH), 126.46 (CH), 125.65 (CH), 125.42 (CH), 125.16 (CH), 121.06 (CH), 105.92 (CH), 55.52 ($CH_3$) ppm. **MS (APCI)** *m/z* (%): 315 (M+1, 100).

**Synthesis of compound 9**

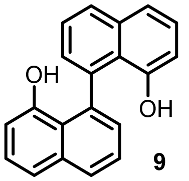


**9**

**Scheme S4.** Chemical structure of compound **9**.

To a solution of compound **8** (75.0 mg, 0.24 mmol) in $CH_2Cl_2$ (2.0 mL) at –78 ºC, $BBr_3$ (80.0 µL, 0.84 mmol) was added dropwise. After the addition, the reaction mixture was allowed to warm to room temperature and stirred for 2 h. The reaction was then quenched by careful addition of water, and the mixture was extracted with $CH_2Cl_2$. The combined organic fractions were washed with water, dried over $Na_2SO_4$, filtered and concentrated under reduced pressure. The crude product was purified by column chromatography ($SiO_2$; hexane:$CH_2Cl_2$ 1:1) to afford compound **9** (65.0 mg, 95%) as a white solid.

**$^1$H NMR** (500 MHz, $CDCl_3$, Fig. S5) δ: 7.98 (dd, *J* = 8.3, 1.3 Hz, 2H), 7.56 (dd, *J* = 8.2, 1.2 Hz, 2H), 7.51 (t, *J* = 7.7 Hz, 2H), 7.44 (t, *J* = 7.9 Hz, 2H), 7.38 (dd, *J* = 7.0, 1.3 Hz, 2H), 6.88 (dd, *J* = 7.6, 1.2 Hz, 2H),

5.39 (s, 2H) ppm. **$^{13}$C NMR** (125 MHz, $CDCl_3$, Fig. S6) δ: 153.36 (C), 135.89 (C), 134.75 (C), 130.21 (CH), 128.90 (CH), 127.89 (CH), 125.01 (CH), 122.11 (C), 121.41 (CH), 112.74 (CH) ppm. **MS (APCI)** *m/z* (%): 286 (M+1, 100).

**Synthesis of DNO**

**DNO**

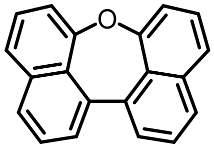

**Scheme S5.** Chemical structure of **DNO**.

Compound **9** (64.0 mg, 0.22 mmol) and *p*-toluenesulfonic acid (63.8 mg, 0.33 mmol) were dissolved in toluene (2.3 mL), and the reaction mixture was refluxed for 3 h. After cooling to room temperature, the solution was quenched with saturated aqueous solution of $K_2CO_3$ and the phases were separated. The organic layer was washed with water, dried over $MgSO_4$, filtered, and concentrated under reduced pressure. The crude solid was recrystallized from *n*-hexane to afford **DNO** (26.0 mg, 43%) as a yellow solid.

**$^{1}$H NMR** (300 MHz, $CDCl_3$, Fig. S7) δ: 8.19 (d, *J* = 7.6 Hz, 1H), 7.80 (d, *J* = 8.0 Hz, 1H), 7.66 (dd, *J* = 6.7, 2.7 Hz, 1H), 7.44 (t, *J* = 7.9 Hz, 2H), 7.38 (dd, *J* = 7.0, 1.3 Hz, 2H), 6.88 (dd, *J* = 7.6, 1.2 Hz, 2H), 7.58 – 7.42 (m, 3H) ppm. **MS (APCI)** *m/z* (%): 268 (M+1, 100).

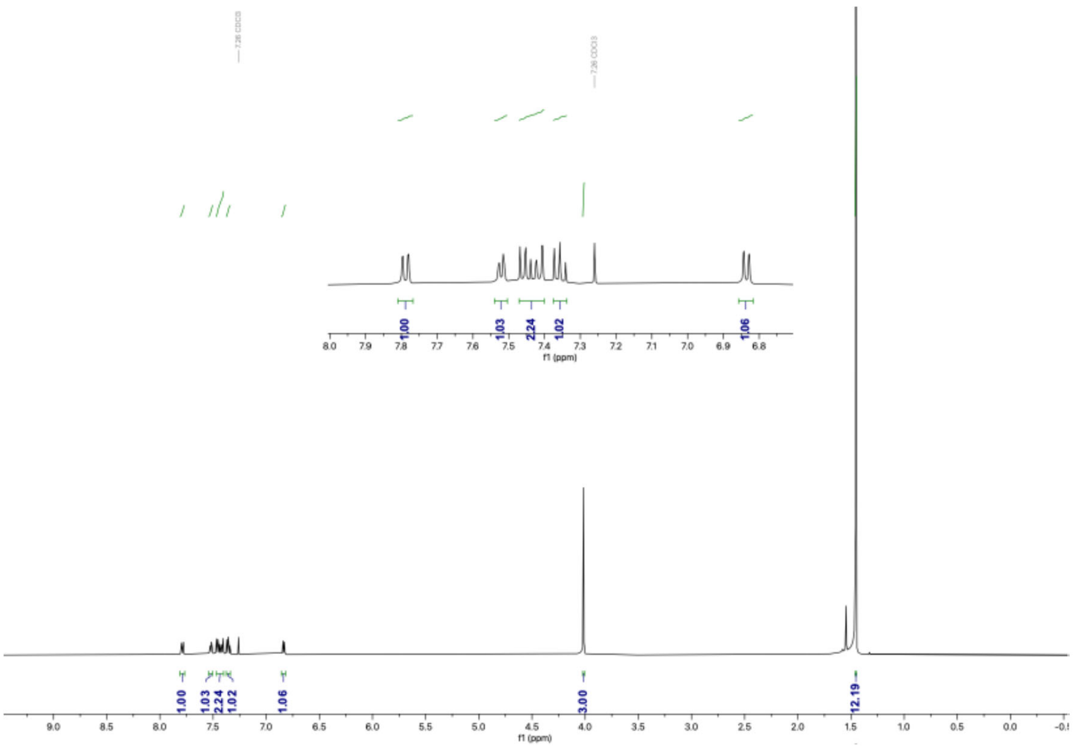

**Figure S1**. $^{1}$H NMR (500 MHz, $CDCl_3$) spectrum of compound **7.**

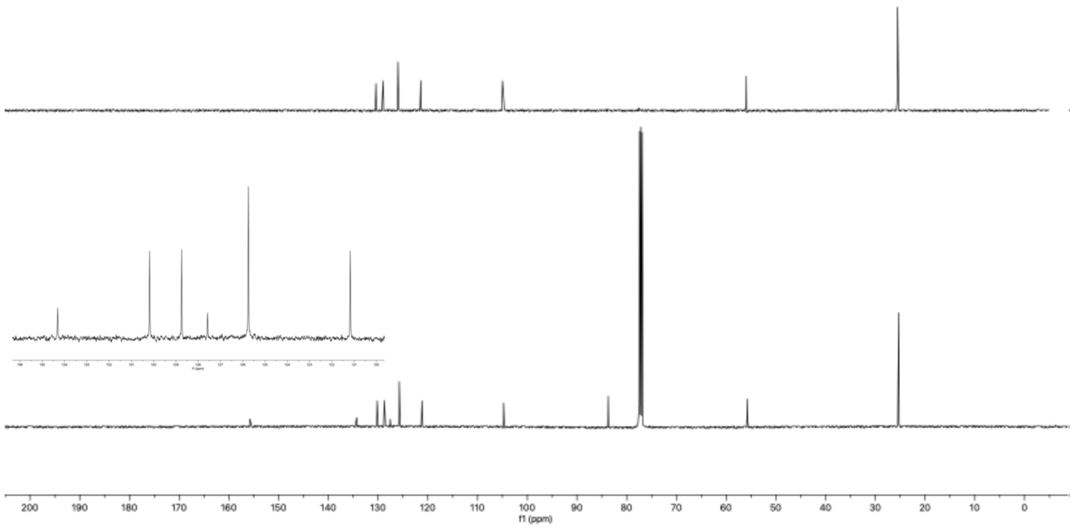

**Figure S2**. $^{13}$C NMR-DEPT (125 MHz, $CDCl_3$) spectrum of compound **7**.

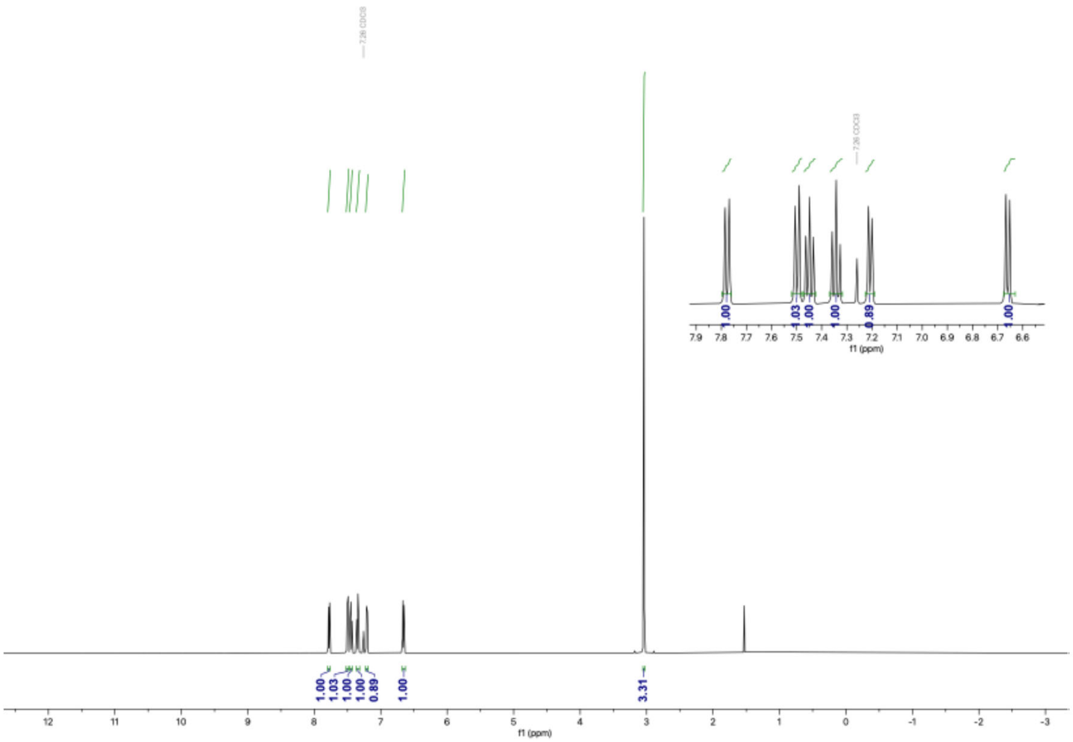


**Figure S3.** $^{1}H$ NMR (500 MHz, $CDCl_3$) spectrum of compound **8.**

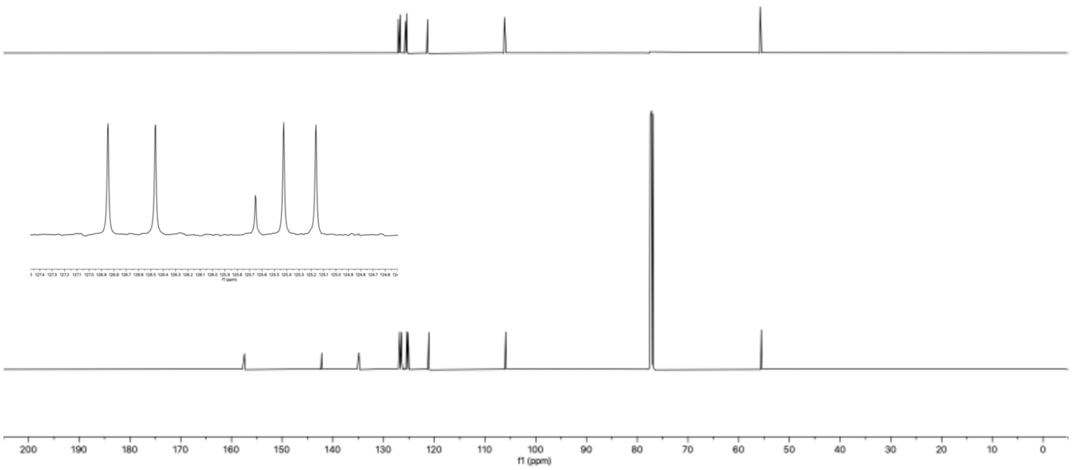


**Figure S4.** $^{13}C$ NMR-DEPT (125 MHz, $CDCl_3$) spectrum of compound **8.**

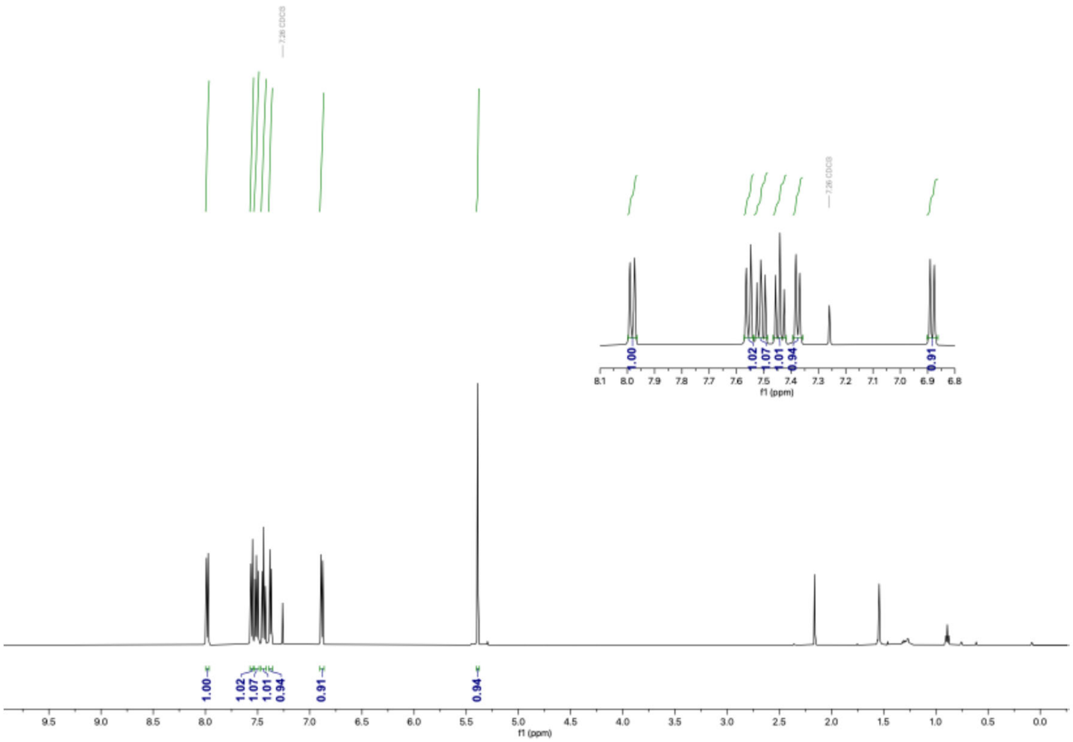

**Figure S5**. $^{1}H$ NMR (500 MHz, $CDCl_3$) spectrum of compound **9.**

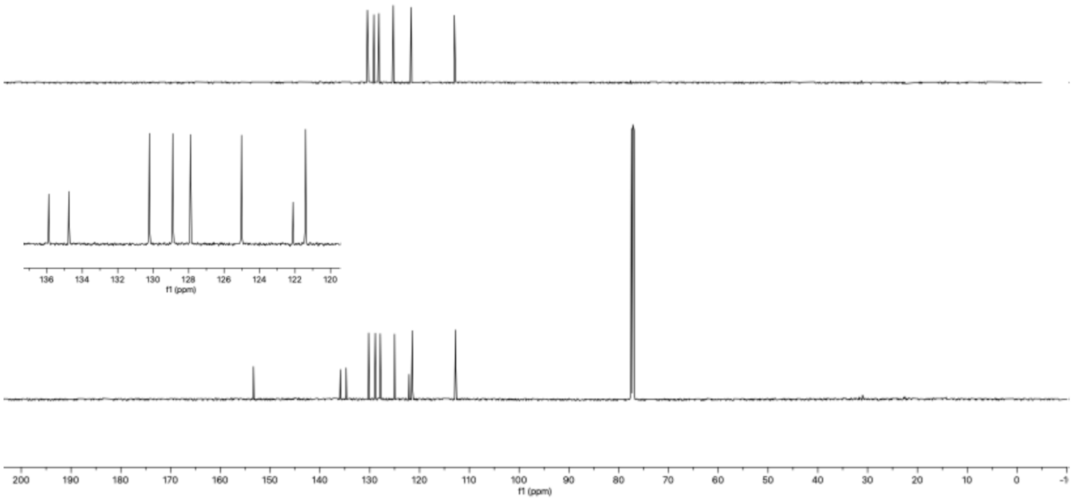

**Figure S6.** $^{13}C$ NMR-DEPT (125 MHz, $CDCl_3$) spectrum of compound **9.**

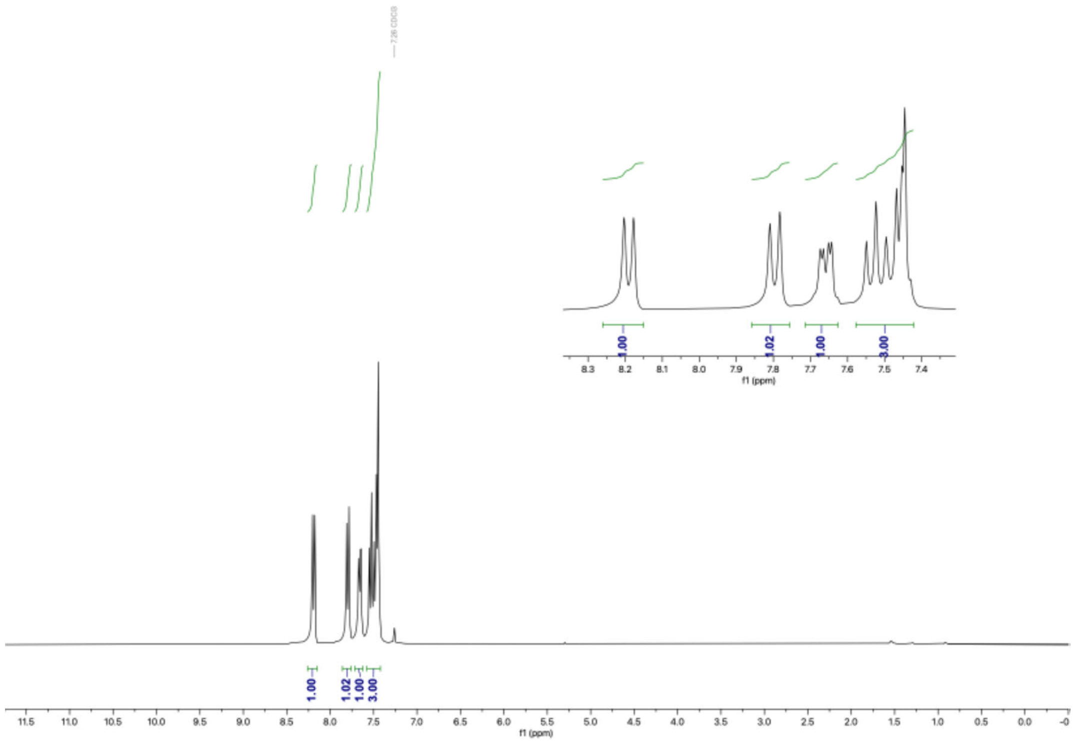


**Figure S7**. $^{1}H$ NMR (300 MHz, $CDCl_3$) spectrum of **DNO**.

### 1.2. Sample preparation and scanning probe microscopy measurements

STM and AFM measurements were performed in a home-built system operating at base pressures below $1 \times 10^{-10}$ mbar and a base temperature of 5 K. Bias voltages were applied to the sample with respect to the tip. All STM and AFM measurements were performed with Cu-coated PtIr tips functionalized with a single carbon monoxide molecule at the tip apex. AFM measurements were performed in non-contact mode with a qPlus sensor.[2] The sensor was operated in frequency-modulation mode[3] with a constant oscillation amplitude of 0.5 Å. STM measurements were performed in constant-current mode, and AFM measurements were performed in constant-height mode with $V = 0$ V. STM and AFM images were post-processed using Gaussian low-pass filters.

The Cu(111) surface was prepared by multiple cycles of sputtering with $Ne^+$ ions and annealing up to 773 K. NaCl was thermally evaporated on the Cu(111) surface held at 283 K. This protocol resulted in the growth of large, defect-free and predominantly bilayer (100)-terminated NaCl films, with a minority of third-layer NaCl islands. The sample quality was ensured by STM imaging before further preparation. Submonolayer coverage of **DNO** on the surface was obtained by flashing an oxidized silicon wafer containing **DNO** molecules in front of the cold sample in the microscope. Carbon monoxide molecules (for tip functionalization) were dosed from the gas phase on the cold sample. The maximum sample temperature during deposition of **DNO** and carbon monoxide molecules was 13 K.

Tip-induced chemistry was performed by relocating the tip at the center of **DNO** molecules at an STM set-point of $V = 0.2$ V and $I = 0.5$ pA. The feedback loop was then opened, and the tip was retracted by 9–11 Å to limit the tunneling current (typically, $I < 50$ pA at the final voltage). The voltage was then ramped from 0.2 V to 4.9–5.1 V in 30 ms and maintained at the final value for 140 ms before ramping back to 0.2 V in 30 ms (Fig. S10). The area was scanned after application of the voltage pulses to monitor the occurrence of reactions. Note that molecules were always found to be displaced on the surface after application of voltage pulses.

### 1.3 DFT calculations

Spin-polarized density functional theory calculations were performed using the Vienna ab initio Simulation Package (VASP).[4–7] The interaction between the core and valence electrons was described using the frozen core projector augmented wave (PAW) method.[8,9] The plane waves were truncated at an energy cutoff of 450 eV in the expansion of the Kohn-Sham orbitals. The considered valence electrons were $1s^1$ (H), $2s^2 2p^2$ (C), $2s^2 2p^4$ (O), $2p^6 3s^1$ (Na), $3s^2 3p^5$ (Cl) and $3d^{10} 4s^1$ (Cu). The exchange-correlation functional was described by the Perdew-Burke-Ernzerhof (PBE) functional.[10] Additional calculations employing the B3LYP functional[11] was used for energy benchmarking. Grimme's D3 correction was included to account for the dispersion interactions for molecules adsorbed on surfaces.[12,13] The electronic structure was regarded as converged when changes in the electronic energy and Kohn-Sham eigenvalues were below $1 \times 10^{-6}$ eV between successive iterations. The structures were optimized using the conjugate gradient method, until the maximum force was below 0.03 eV/Å.

The lateral lattice constant of a bilayer NaCl was determined to be 5.526 Å using a $(\sqrt{2} \times \sqrt{2})$R45° surface cell (composed of 4 Na and 4 Cl atoms). The lattice constant of bulk Cu was determined to be 3.57 Å (with D3 correction), which is slightly lower than the experimental value of 3.61 Å.[14] Gas-phase species were optimized using a (32, 31, 30) Å vacuum box. Transition state calculations were performed using the climbing-image nudged elastic band method[15,16] using a (19, 18, 15) Å vacuum box. Structural optimization and nudged elastic band calculation for anionic species were performed by adding an excess electron to the system. Vibrational analyses were performed assuming the harmonic approximation and solved using finite difference to confirm the transition states.

The charge state of the system was analyzed using Bader charge analyses.[17] Molecular orbitals of gas-phase molecules were calculated using Dmol[3] (ref.[18]).

The investigated surface was composed of a bilayer of $(5\sqrt{2} \times 4\sqrt{2})$R45° NaCl(100) supported on $(11 \times 5\sqrt{3})$rect. Cu(111). The optimized lattice constant for the bilayer of NaCl was used, whereas the Cu surface was strained to match the lattice of NaCl. The strains on Cu(111) were –0.50 % and 1.11 % in the two dimensions, respectively. The surface is shown in Fig. S8. In the calculations, the bottom layer of the strained Cu(111) was kept fixed to emulate a bulk system.

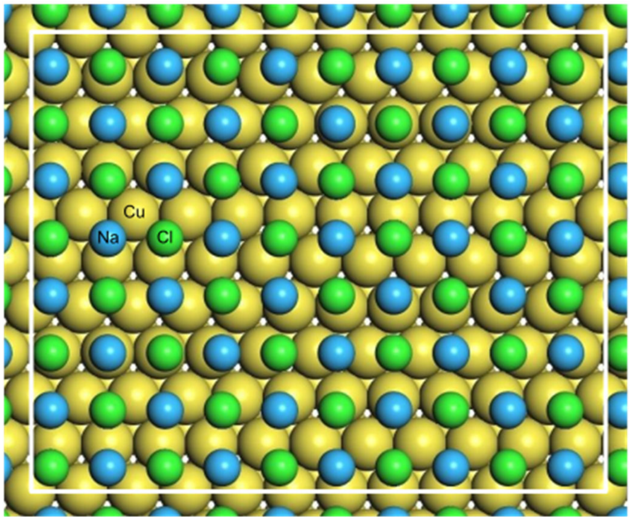


**Figure S8.** Ball model of the investigated bilayer NaCl/Cu(111) system. Atomic color codes: blue (Na), green (Cl), and yellow (Cu).

The main approximation in the DFT calculations is the choice of the exchange-correlation functional. The applied PBE functional is generally a good compromise when having systems with molecules adsorbed on surfaces. However, PBE is known to over-delocalize the electron density, which could influence the energetic preferences. B3LYP is a computationally expensive hybrid functional that reduces the over-delocalization issue. We performed a set of benchmark calculations with the B3LYP functional. The energy difference between **DNO** and **Int3** is –1.16 eV with PBE and –1.11 eV with B3LYP. The energy difference between **Int3** and (Compound **2** + OH) is 4.81 eV with PBE and 4.48 eV with B3LYP. Thus, the trends between the two functionals are consistent.

## 2. Supporting data

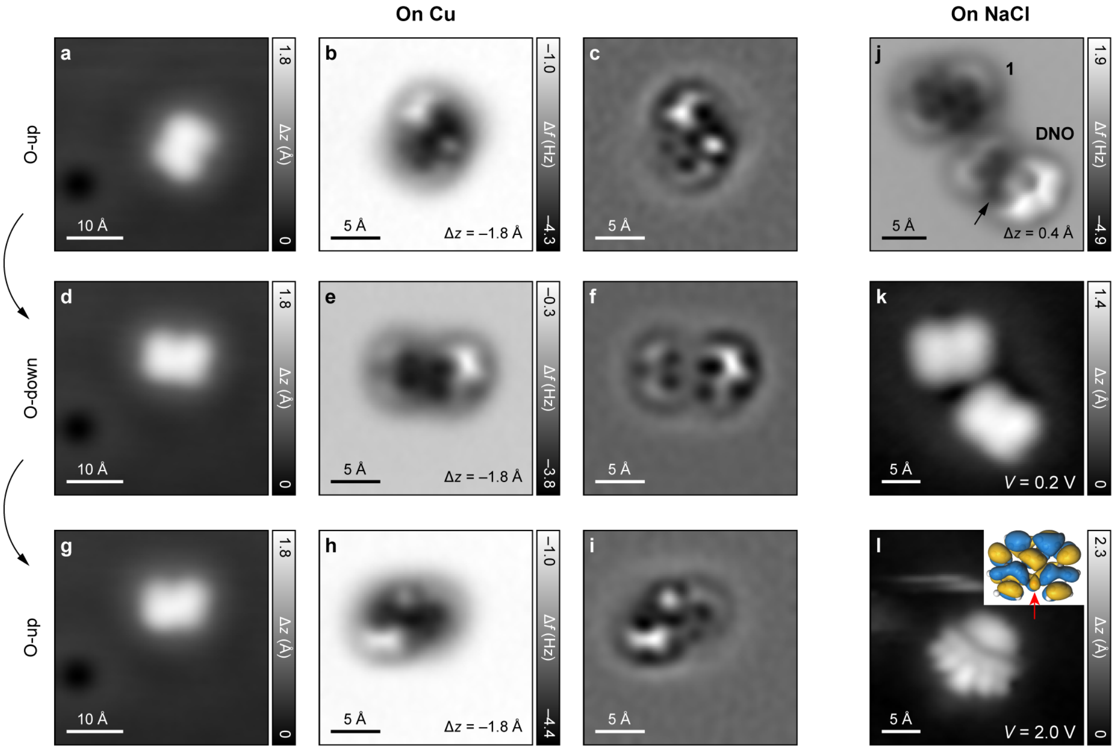


**Fig. S9.** Additional measurements on **DNO**. (a–c) STM (a), AFM (b) and corresponding Laplace-filtered AFM (c) images of an O-up **DNO** molecule on Cu. The oxygen atom appears as a bright protrusion in AFM imaging, as for an O-up **DNO** molecule on NaCl (Fig. 2). (d–f) STM (d) and AFM (e, f) images of an O-down **DNO** molecule on Cu, obtained after application of a voltage pulse of 4.9 V to the O-up molecule in (a–c). The oxygen atom in the O-down conformation is not visible in AFM imaging, as for an O-down **DNO** molecule on NaCl (j and Fig. 2). On further scanning the O-down molecule, its conformation changed back to O-up, as shown in the STM and AFM images in (g–i). (j, k) AFM (j) and STM (k) images of a **1**⁻ and an O-down **DNO** molecule adsorbed next to each other (also shown in Fig. 2). (l) Corresponding STM image showing the LUMO density of the **DNO** molecule. The DFT calculated LUMO of **DNO** is also shown (isosurface: $0.01a_0^{-3/2}$, $a_0$ denotes the Bohr radius). At this elevated voltage, the **1**⁻ molecule was mobile and could not be imaged stably (resulting in the streaks in the image). The arrows in (j, l) indicate the location of the oxygen atom. Scanning parameters for STM images: *V* = 0.2 V, *I* = 0.5 pA (a, d, g); *I* = 0.3 pA (k) and *I* = 0.2 pA (l). Open feedback parameters for AFM images: *V* = 0.2 V, *I* = 0.5 pA on Cu (b, c, e, f, h, i) and NaCl (j).

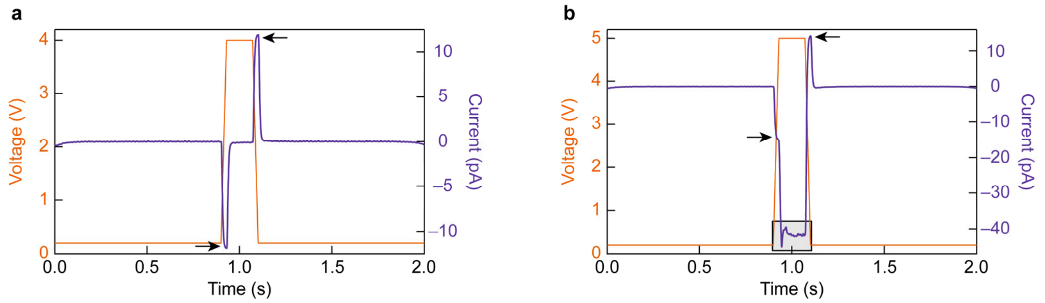


**Figure S10.** Representative current and voltage traces during application of voltage pulses to **DNO** molecules. See section 1.2 for details. (a) Voltage pulse of 4 V with the tip retracted by 10 Å, which did not result in any reaction. The arrows in (a, b) denote features in the current signal that result from capacitive coupling of the voltage ramp to the measured current signal. (b) Voltage pulse of 5 V with the tip retracted by 9 Å, which resulted in the generation of perylene. The shaded box denotes changes in the current that result from both the oxygen deletion reaction and displacement of the molecule on the NaCl surface. The data in (a, b) were acquired with the same tip.

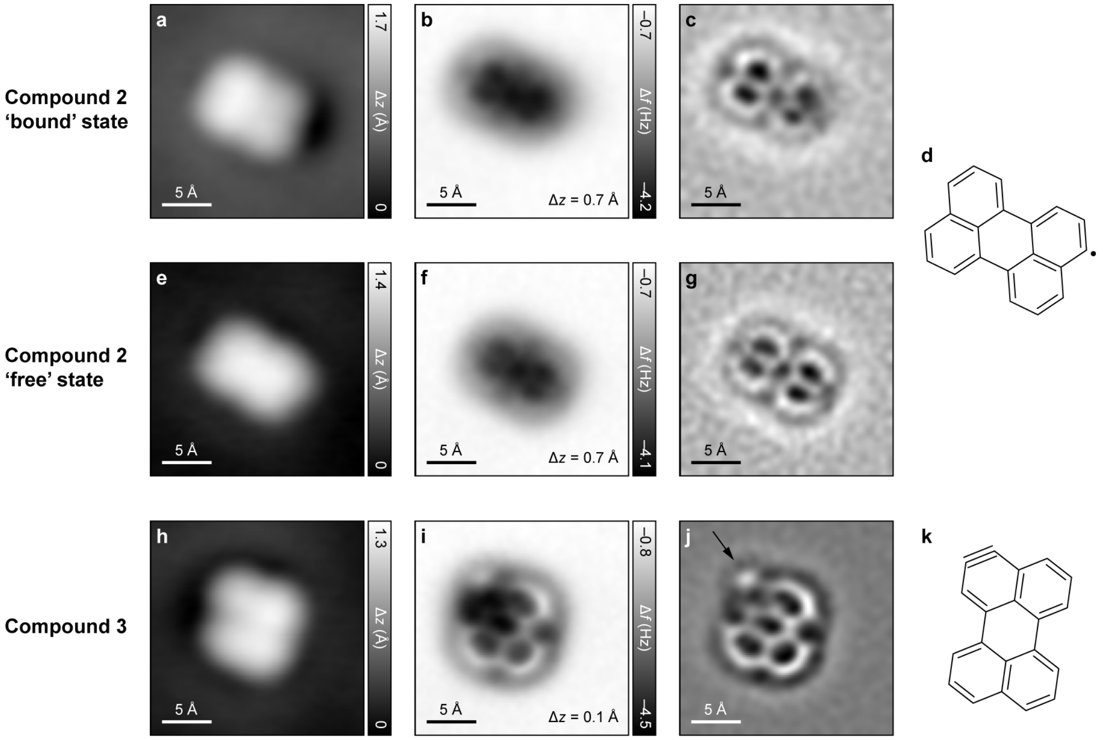


**Fig. S11.** Imaging of compounds **2** and **3**. (a–g) STM and AFM images of a perylenyl radical $C_{20}H_{11}$ (**2**), observed in 2/21 cases. Note that in perylenyl radical, the σ radical may be located at one of the three inequivalent ortho (2, 5, 8, 11 positions), peri (3, 4, 9, 10 positions) or bay (1, 6, 7, 12 positions) sites of perylene. For simplicity, we use the label **2** to denote species with the radical at any of the three sites. We observed **2** to exist in 'free' (highly mobile on the surface) and 'bound' (comparatively less mobile) states on NaCl, as previously observed by Zhong et al. for other polycyclic conjugated hydrocarbon σ radicals.[19] (a, e) STM images of **2** in the bound (a) and free (e) states. (b, f) Corresponding AFM images of **2** in the bound (b) and free (f) states. (c, g) Laplace-filtered versions of b (c) and f (g). The data in (a–g) were acquired on the same molecule with the same tip. The chemical structure of **2** corresponding

to (a–c) and (e–g) is shown in (d). (h–j) STM (h), AFM (i) and corresponding Laplace-filtered AFM (j) images of didehydroperylene $C_{20}H_{10}$ (**3**), observed in 1/21 cases. The bright feature in the upper left benzenoid ring, indicated by an arrow in (j), is related to the C–C triple bond.[20,21] (k) Chemical structure of **3**. Scanning parameters for STM images: $V$ = 0.2 V, $I$ = 0.3 pA (a, e) and 0.5 pA (h). Open feedback parameters for AFM images: $V$ = 0.2 V, $I$ = 0.5 pA on NaCl.

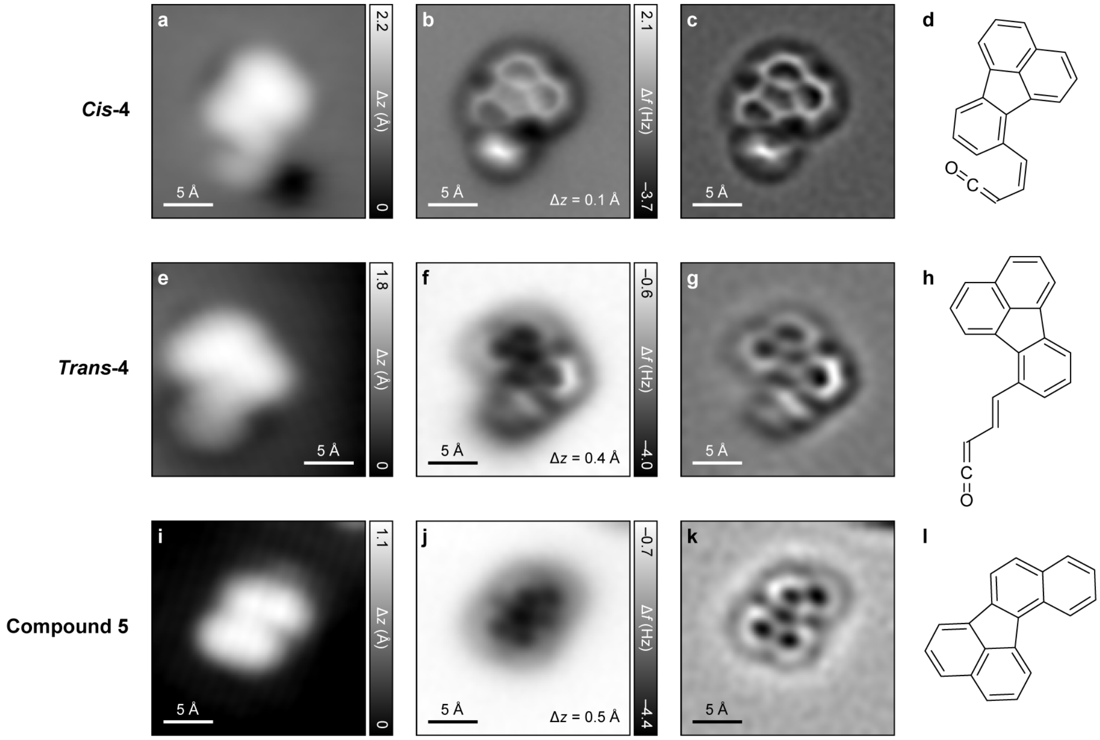


**Fig. S12.** Imaging of compounds **4** and **5**. (a–h) STM and AFM images of *cis* (a–d) and *trans* (e–h) isomers of **4** (observed in 4/21 cases), resulting from ring opening reactions of **DNO**. (a, e) STM images of *cis*- (a) and *trans*- (e) **4**. (b, f) Corresponding AFM images of *cis*- (b) and *trans*- (f) **4**. (c, g) Laplace-filtered versions of b (c) and f (g). (d, h) Tentative chemical structures of *cis*- (d) and *trans*- (h) **4**. (i–k) STM (i), AFM (j) and corresponding Laplace-filtered AFM (k) images of **5** (observed in 1/21 cases). (l) Chemical structure of **5**. Scanning parameters for STM images: $V$ = 0.2 V, $I$ = 0.5 pA. Open feedback parameters for AFM images: $V$ = 0.2 V, $I$ = 0.5 pA on NaCl.

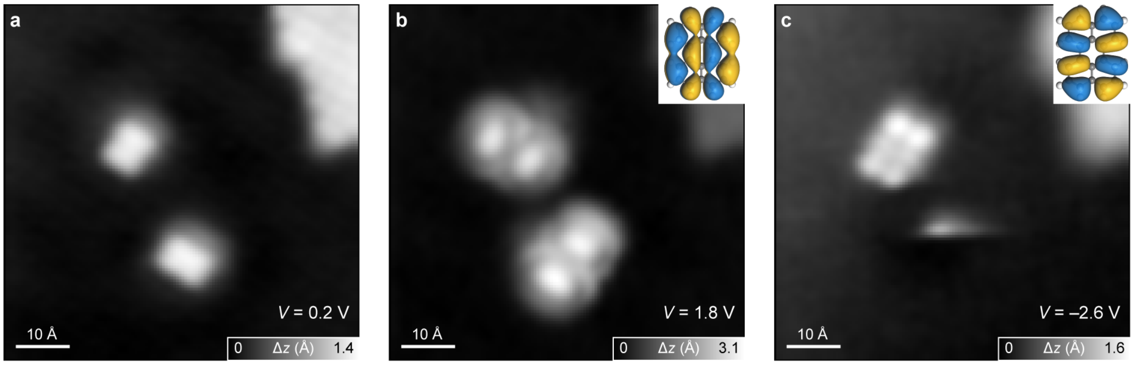


**Fig. S13.** Orbital density imaging of perylene. (a) In-gap STM image of two perylene molecules on NaCl. The upper right corner of the scan frame contains a third-layer NaCl island. (b, c) Corresponding STM images at the voltages indicated in the respective panels, showing the LUMO (b) and HOMO (c) density of perylene. The DFT calculated LUMO and HOMO of perylene are also shown in (b) and (c), respectively (isosurface: $0.01a_0^{-3/2}$). The elevated voltage in (c) led to movement of one of the perylene molecules. Scanning parameters: $I$ = 0.50 pA (a), $I$ = 0.15 pA (b) and $I$ = 0.12 pA (c).

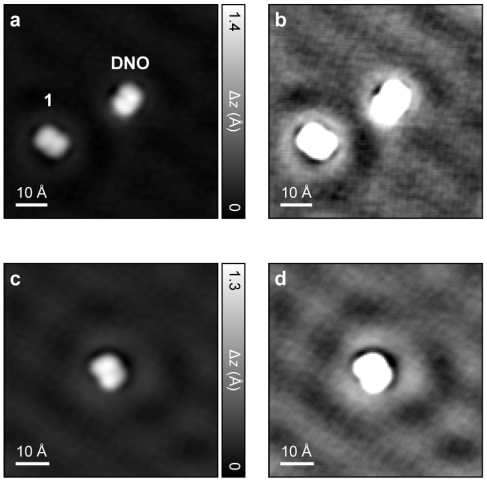


**Fig. S14.** Observation of NaCl/Cu(111) interface-state scattering by compound **1**. (a, b) STM image of **1** and **DNO** shown with two contrast levels. Strong scattering of the NaCl/Cu(111) interface state[22,23] is observed by **1**, supporting its charged state, while **DNO**, which is neutral, does not scatter the interface state strongly. (c, d) STM image of **1** shown with two contrast levels. Concentric ring-like features are visible around **1** due to interface-state scattering. Scanning parameters: $V$ = 0.2 V (a, b) and $V$ = 0.1 V (c, d), $I$ = 0.5 pA. The voltages in (a–d) are chosen to lie above the onset[22] of NaCl/Cu(111) interface state (at $V$ ~ –0.2 V).

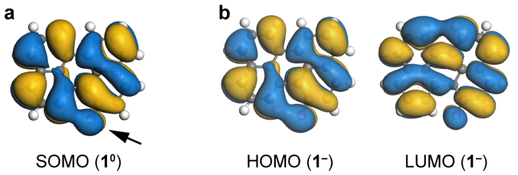


**Fig. S15.** The DFT calculated singly occupied molecular orbital (SOMO) of $\mathbf{1}^0$ (a), and HOMO (left) and LUMO (right) of $\mathbf{1}^-$ (b) (isosurface: $0.01a_0^{-3/2}$). $\mathbf{1}^0$ is an open-shell molecule with one unpaired π-electron, whereas $\mathbf{1}^-$ is a closed-shell molecule. The arrow in (a) indicates the oxygen atom.

**Table S1.** Optimized C–O bond lengths of selected molecules from DFT calculations.

| **Molecule** | **Charge state** | **C–O bond length (Å)** |
|---|---|---|
| Compound **1** 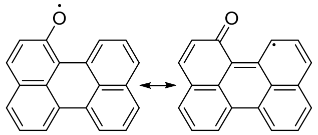 | Neutral, in gas phase<br>Anionic, in gas phase<br>Anionic, on bilayer NaCl/Cu(111) | 1.26<br>1.27<br>1.29 |
| **Int3** 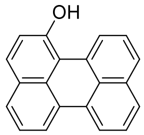 | Neutral, in gas phase<br>Anionic, in gas phase<br>Neutral, on bilayer NaCl/Cu(111) | 1.38<br>1.40<br>1.37 |
| Cyclohexanone 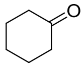 | Neutral, in gas phase<br>Anionic, in gas phase | 1.23<br>1.25 |
| Phenol 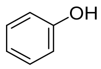 | Neutral, in gas phase<br>Anionic, in gas phase | 1.38<br>1.37 |

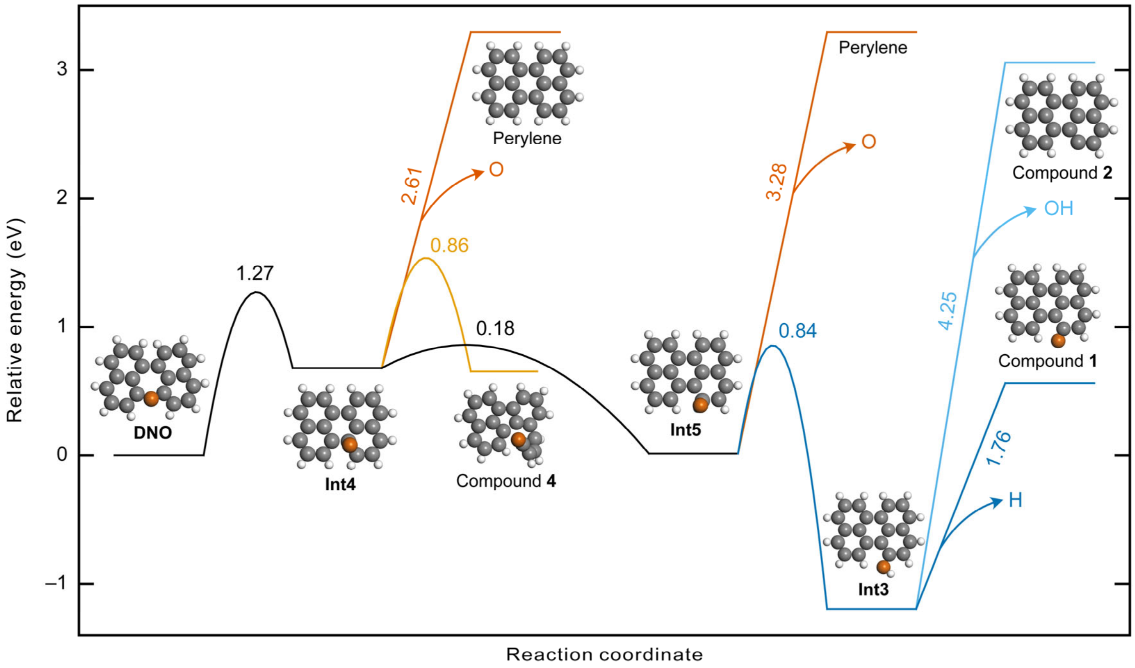


**Fig. S16.** Calculated gas-phase potential energy landscape for skeletal editing of **DNO**, assuming a global anionic state; that is, anionic states of the reactant, intermediates and products. The optimized gas-phase geometries of the molecular species are also shown. The numbers adjacent to the curves denote activation energies in eV. Note that for the reactions **Int4** and **Int5** → perylene and **Int3** → compounds **1** and **2**, energy differences coincide with activation energies. All activation energies are lowered compared to the neutral case (Fig. 3 in the main text). In the intermediates **Int4** and **Int5**, the oxygen atom is located on top of a carbon atom. For the neutral case, the corresponding intermediates **Int1** and **Int2** have the oxygen atom located in a C–C bridge site (Fig. 3). Note that from our experiments, we cannot deduce whether the reactions occur in the neutral or anionic charge state. Compound **1**, which is found in an anionic charge state on bilayer NaCl/Cu(111), might be charged by electron transfer from the surface after it is formed.

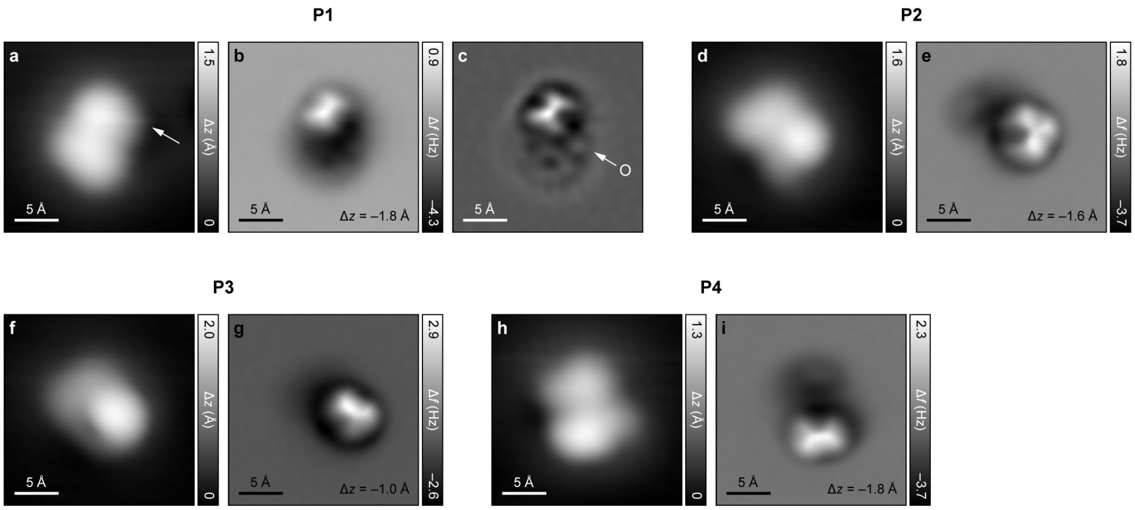


**Fig. S17.** Reaction products on Cu(111). STM and AFM images of four products **P1–P4** obtained after application of voltage pulses of $V = 5.0$ V (**P1**–**P3**) and $V = 4.9$ V (**P2**) to individual **DNO** molecules. (a–

c) STM (a), AFM (b) and Laplace-filtered AFM (c) images of **P1**, which still contains the oxygen atom indicated by the arrow in (c). The benzenoid ring, whose location is indicated by the arrow in (a), has likely lost one or more hydrogen atoms via cleavage of C($sp^2$)–H bonds. This ring is not resolved in AFM imaging (b, c) likely due to the formation of C–Cu bonds, which leads to bending of the ring toward the surface. (d–i) STM (d, f, h) and AFM (e, g, i) images of **P2**–**P4**, which are difficult to identify. It is likely that **P2**–**P4** exhibit loss of one or more hydrogen atoms. However, it is unclear if the oxygen atom is present in these molecules. Scanning parameters for STM images: $V$ = 0.2 V, $I$ = 0.5 pA. Open feedback parameters for AFM images: $V$ = 0.2 V, $I$ = 0.5 pA on Cu.

# 3. References

(1) Dobelmann, L.; Parham, A. H.; Büsing, A.; Buchholz, H.; König, B. First Synthesis of Naphthalene Annulated Oxepins. *RSC Adv.* **2014**, *4* (105), 60473–60477. https://doi.org/10.1039/C4RA10652K.
(2) Giessibl, F. J. High-Speed Force Sensor for Force Microscopy and Profilometry Utilizing a Quartz Tuning Fork. *Appl. Phys. Lett.* **1998**, *73* (26), 3956–3958.
(3) Albrecht, T. R.; Grütter, P.; Horne, D.; Rugar, D. Frequency Modulation Detection Using high-Q Cantilevers for Enhanced Force Microscope Sensitivity. *J. Appl. Phys.* **1991**, *69* (2), 668–673.
(4) Kresse, G.; Hafner, J. Ab Initio Molecular Dynamics for Liquid Metals. *Phys. Rev. B* **1993**, *47* (1), 558–561.
(5) Kresse, G.; Hafner, J. Ab Initio Molecular Dynamics for Open-Shell Transition Metals. *Phys. Rev. B* **1993**, *48* (17), 13115–13118.
(6) Kresse, G.; Hafner, J. Ab Initio Molecular-Dynamics Simulation of the Liquid-Metal--Amorphous-Semiconductor Transition in Germanium. *Phys. Rev. B* **1994**, *49* (20), 14251–14269.
(7) Kresse, G.; Furthmüller, J. Efficient Iterative Schemes for Ab Initio Total-Energy Calculations Using a Plane-Wave Basis Set. *Phys. Rev. B* **1996**, *54* (16), 11169–11186.
(8) Blöchl, P. E. Projector Augmented-Wave Method. *Phys. Rev. B* **1994**, *50* (24), 17953–17979.
(9) Kresse, G.; Joubert, D. From Ultrasoft Pseudopotentials to the Projector Augmented-Wave Method. *Phys. Rev. B* **1999**, *59* (3), 1758–1775.
(10) Perdew, J. P.; Burke, K.; Ernzerhof, M. Generalized Gradient Approximation Made Simple. *Phys. Rev. Lett.* **1996**, *77* (18), 3865–3868.
(11) Stephens, P. J.; Devlin, F. J.; Chabalowski, C. F.; Frisch, M. J. Ab Initio Calculation of Vibrational Absorption and Circular Dichroism Spectra Using Density Functional Force Fields. *J. Phys. Chem.* **1994**, *98* (45), 11623–11627.
(12) Grimme, S.; Antony, J.; Ehrlich, S.; Krieg, H. A Consistent and Accurate Ab Initio Parametrization of Density Functional Dispersion Correction (DFT-D) for the 94 Elements H-Pu. *J. Chem. Phys.* **2010**, *132* (15), 154104.
(13) Grimme, S.; Ehrlich, S.; Goerigk, L. Effect of the Damping Function in Dispersion Corrected Density Functional Theory. *J. Comput. Chem.* **2011**, *32* (7), 1456–1465.
(14) Crystal Structures and Lattice Parameters of Allotropes of the Elements. In *CRC Handbook of Chemistry and Physics*; Rumble, J. R., Ed.; 104th ed.; CRC Press, Inc., 2023.
(15) Mills, G.; Jónsson, H.; Schenter, G. K. Reversible Work Transition State Theory: Application to Dissociative Adsorption of Hydrogen. *Surf. Sci.* **1995**, *324* (2), 305–337.
(16) Henkelman, G.; Uberuaga, B. P.; Jónsson, H. A Climbing Image Nudged Elastic Band Method for Finding Saddle Points and Minimum Energy Paths. *J. Chem. Phys.* **2000**, *113* (22), 9901–9904.
(17) Henkelman, G.; Arnaldsson, A.; Jónsson, H. A Fast and Robust Algorithm for Bader Decomposition of Charge Density. *Comput. Mater. Sci.* **2006**, *36* (3), 354–360.
(18) Delley, B. From Molecules to Solids with the $DMol^3$ Approach. *J. Chem. Phys.* **2000**, *113* (18), 7756–7764.
(19) Zhong, Q.; Ihle, A.; Ahles, S.; Wegner, H. A.; Schirmeisen, A.; Ebeling, D. Constructing Covalent Organic Nanoarchitectures Molecule by Molecule via Scanning Probe Manipulation. *Nat. Chem.* **2021**, *13* (11), 1133–1139.
(20) Gross, L.; Mohn, F.; Moll, N.; Schuler, B.; Criado, A.; Guitián, E.; Peña, D.; Gourdon, A.; Meyer, G. Bond-Order Discrimination by Atomic Force Microscopy. *Science* **2012**, *337* (6100), 1326–1329.
(21) de Oteyza, D. G.; Gorman, P.; Chen, Y.-C.; Wickenburg, S.; Riss, A.; Mowbray, D. J.; Etkin, G.; Pedramrazi, Z.; Tsai, H.-Z.; Rubio, A.; Crommie, M. F.; Fischer, F. R. Direct Imaging of Covalent Bond Structure in Single-Molecule Chemical Reactions. *Science* **2013**, *340* (6139), 1434–1437.
(22) Repp, J.; Meyer, G.; Rieder, K.-H. Snell's Law for Surface Electrons: Refraction of an Electron Gas Imaged in Real Space. *Phys. Rev. Lett.* **2004**, *92* (3), 036803.
(23) Swart, I.; Sonnleitner, T.; Repp, J. Charge State Control of Molecules Reveals Modification of the Tunneling Barrier with Intramolecular Contrast. *Nano Lett.* **2011**, *11* (4), 1580–1584.
